# An Internet-Based Cognitive Behavioral Therapy Program for Anxiety and Depression (Tranquility): Adaptation Co-design and Fidelity Evaluation Study

**DOI:** 10.2196/33374

**Published:** 2022-02-02

**Authors:** Victoria C Patterson, Meghan A Rossi, Alissa Pencer, Lori Wozney

**Affiliations:** 1 Department of Psychology and Neuroscience Dalhousie University Halifax, NS Canada; 2 Nova Scotia Health Halifax, NS Canada

**Keywords:** cognitive behavioral therapy, anxiety, depression, fidelity, usability

## Abstract

**Background:**

Internet-based cognitive behavioral therapy (iCBT) is a necessary step toward increasing the accessibility of mental health services. Yet, few iCBT programs have been evaluated for their fidelity to the therapeutic principles of cognitive behavioral therapy (CBT) or usability standards. In addition, many existing iCBT programs do not include treatments targeting both anxiety and depression, which are commonly co-occurring conditions.

**Objective:**

This study aims to evaluate the usability of Tranquility—a novel iCBT program for anxiety—and its fidelity to CBT principles. This study also aims to engage in a co-design process to adapt Tranquility to include treatment elements for depression.

**Methods:**

CBT experts (n=6) and mental health–informed peers (n=6) reviewed the iCBT program Tranquility. CBT experts assessed Tranquility’s fidelity to CBT principles and were asked to identify necessary interventions for depression by using 2 simulated client case examples. Mental health–informed peers engaged in 2 co-design focus groups to discuss adaptations to the existing anxiety program and the integration of interventions for depression. Both groups completed web-based surveys assessing the usability of Tranquility and the likelihood that they would recommend the program.

**Results:**

The CBT experts’ mean rating of Tranquility’s fidelity to CBT principles was 91%, indicating a high fidelity to CBT. Further, 5 out of 6 CBT experts and all mental health–informed peers (all participants: 11/12, 88%) rated Tranquility as satisfactory, indicating that they may recommend Tranquility to others, and they rated its usability highly (mean 76.56, SD 14.07). Mental health–informed peers provided suggestions on how to leverage engagement with Tranquility (eg, adding incentives and notification control).

**Conclusions:**

This preliminary study demonstrated the strong fidelity of Tranquility to CBT and usability standards. The results highlight the importance of involving stakeholders in the co-design process and future opportunities to increase engagement.

## Introduction

### Background

The in-person delivery of cognitive behavioral therapy (CBT) is an effective, evidence-based approach for treating a wide range of mental health conditions with a substantial amount of research indicating its efficacy for the treatment of anxiety [1] and depression [2]. In Canada, mild to moderate depression and anxiety disorders affect up to 11.7% and 10.8% of the adult population, respectively [3]. Although these mental health challenges affect many Canadians, there are numerous barriers to accessing evidence-based treatment. The availability of treatment is hindered by lengthy wait lists, therapists’ caseload limitations, and the moderate to low availability of clinicians trained in the delivery of evidence-based CBT [4]. The accessibility of treatment is further hindered by the monetary costs of treatment outside the public system and geographic access for those living in rural areas [5]. During the COVID-19 pandemic, additional challenges to accessing in-person treatment included physical distancing and self-isolation measures related to COVID-19, in addition to the sharp increase in stress, anxiety, and depression associated with the pandemic [6,7].

Previous research has found that internet-based CBT (iCBT) interventions provide increased access to evidence-based treatment, which can improve the mental health of the general population by effectively reducing symptoms associated with anxiety and depression [8,9]. However, few web-based interventions have been reviewed by therapy experts and have demonstrated treatment fidelity to core evidence-based CBT treatment protocols. Treatment fidelity is defined as “the extent to which an intervention is delivered as intended by the protocol” [10,11]. For an iCBT program targeting anxiety and depression, treatment fidelity indicates how closely the program follows the standard CBT approach for these mental health challenges and whether the necessary therapeutic interventions (eg, exposure and behavioral activation) are included. Bowie-DaBreo et al [12] described how there is significant uncertainty about the effectiveness of many mental health apps and programs, and much of that uncertainty appears to stem from a lack of information about treatment fidelity. In addition, few programs appeared to follow the guidelines for evidence-based care for specific challenges (eg, anxiety). Furthermore, a recent study examining mobile apps to treat depression [13] found that few programs followed a CBT approach, even though the literature supports CBT as the gold standard for the treatment of anxiety and depression [14]. In 2018, an international group of researchers developed a list of recommendations for the development of iCBT programs [15]—one of their core recommendations was the use of a co-design process (ie, involvement of intended users in the development of the iCBT intervention). Co-design has many benefits (eg, improved program tailoring) [16], yet this approach appears underused in the literature. In sum, there appears to be a gap in the literature pertaining to evidence-based iCBT programs that also follow the iCBT recommendations for development, especially for programs targeting co-occurring depression and anxiety [17].

### Tranquility Program

Tranquility was designed to be an iCBT intervention that increases cognitive and behavioral skills (eg, thought challenging, exposure, and behavioral experiments) of individuals with mild to moderate anxiety. Users learn ways to manage their symptoms in addition to gaining access to personalized support through video, phone, and in-app messaging with a web-based coach. Results from a pilot program evaluation of Tranquility illustrated decreases in anxiety and stress levels in individuals who completed 3 or more modules, meaning that people who engaged with the program were noted to benefit, and most program users found the program to be helpful [18,19]. Though designed by a team including licensed psychologists with advanced skills in CBT techniques, researchers, and people with lived experience, Tranquility has not yet undergone a rigorous evaluation of its fidelity to core CBT components linked to improved client outcomes by a broader team of experts in this field. Furthermore, engaging mental health–informed peers (eg, people with lived experience of mental health challenges or peer counselors) in program development and evaluation could further illuminate usability characteristics (ie, aspects that contribute to the technical effectiveness and efficiency of a program) [20,21] that enable and increase engagement. Finally, Tranquility has the potential to be adapted for dual treatment streams related to anxiety and depression but requires both clinical and first voice input to map development.

### Study Aims

This study aims to address these gaps by (1) evaluating Tranquility’s fidelity to CBT principles; (2) assessing CBT experts’ and mental health–informed peers’ perceptions of the usability of the Tranquility anxiety program and the likelihood they would recommend it to others; and (3) co-designing an adaptation of the Tranquility program to include treatment for depression with a group of mental health–informed peers.

## Methods

### Recruitment

To recruit focus group participants, relevant networks (eg, local university programs and peer counseling programs) were contacted and asked to disseminate study information to all local individuals on their newsletter mailing list. The focus group participants had to identify as a first voice advocate or have experience with mental health conditions in a near-peer role where they would have advanced knowledge of needs and experiences of the population for which Tranquility was designed (eg, peer mentors on university campuses). CBT experts were recruited through the authors’ professional networks of CBT clinicians and North American CBT organizations (eg, Canadian Association of Cognitive and Behavioural Therapies and Association for Behavioral and Cognitive Therapies), and individuals who met the study criteria were contacted directly via email. CBT experts had to have 5 years of experience delivering CBT for depression and anxiety in adults and must be licensed by a professional body. All participants were required to be ≥18 years of age.

We recruited 6 mental health–informed peers, referred to as focus group participants throughout the remainder of the paper, and 6 CBT experts to participate in the study. Sociodemographic information of all participants is presented in Table 1. Across both groups, participants were predominately White, heterosexual women. The mean age of the focus group was 39.25 (SD 8.88) years, and the mean age of experts was 47.6 (SD 14.72) years. CBT experts were entered into a draw to win a web-based gift card, while the focus group participants received an honorarium following each focus group. The study procedures were approved by the Research Ethics Board of the Nova Scotia Health Authority (file number 1025561) and conformed to the ethical standards of research set out by the Canadian Tri-Council Policy Statement 2 [22].

**Table 1:** Participant demographics.

Demographics			Focus group participants (n=6)		CBT^a^ experts (n=6)
Age (years), mean (SD)			39.25 (8.88)		47.6 (14.72)
**Gender, n (%)^b^**					
	Man	—^c^		1 (17)	
	Woman	5 (83)		5 (83)	
	Preferred not to say	1 (17)		—	
**Sexual orientation, n (%)^b^**					
	Heterosexual	4 (66)		6 (100)	
	Bisexual	1 (17)		—	
	Preferred not to say	1 (17)		—	
**Race, n (%)^b^**					
	White	5 (83)		6 (100)	
	Preferred not to say	1 (17)		—	

^a^CBT: cognitive behavioral therapy.

^b^Participants were provided with a comprehensive list of gender identities, sexual orientations, and racial identities from which to choose when reporting their demographic information—only those selected by at least 1 participant are listed in this table.

^c^Not available. These categories were not used by the focus group or cognitive behavioral therapy experts.

### Measures

#### Focus Groups

##### Overview

Mental health–informed peers were involved in 2 focus groups to co-design adaptations to Tranquility for both anxiety and depression and the benefit of these changes. A script with a combination of question types (eg, open-ended and close-ended questions) for the focus group was developed by the research team and can be found in Multimedia Appendix 1. The ECOUTER (Employing Conceptual Schema for Policy and Translation Engagement in Research) [23] framework was used in the facilitation of the focus groups. This framework positions participants in a prominent role in the research process by encouraging knowledge exchange, developing a conceptual schema, analyzing discussion contributions, and refining recommendations. Following each focus group, participants completed a web-based survey, which included demographic questions (eg, age and gender), the usability of the web-based platform, and their likelihood of recommending Tranquility to others.

##### The System Usability Scale

The System Usability Scale (SUS) [24] is a validated 10-item scale that can be adapted to assess facets of usability in different programs (eg, “I thought that the Tranquility program was easy to use”). Items are rated on a 5-point scale ranging from *strongly disagree* (score=1) to *strongly agree* (score=5). To obtain an overall usability score, a value of 1 is subtracted from the score of odd-numbered items, and the respondent’s score is subtracted from 5 for each even-numbered question. These new values for each item are summed, and the total is multiplied by 2.5 to create scores ranging from 0 to 100. Scores >69 reflect appropriate ratings of usability, with higher scores indicating greater usability [25]. Scores in the high 70s to 80s indicate good usability, while scores ≥90 indicate high overall usability. The SUS is the most widely used measure that is validated for testing usability across various iCBT programs [26,27], and it is designed to be tailored to the program being tested.

##### The Likelihood to Recommend Scale

Participants rated how likely they were to recommend Tranquility via a 1-item measure: “How likely are you to recommend Tranquility to your friends, family, or associates?” [28]. Responses were rated on an 11-point scale ranging from *not at all likely* (score=0) to *extremely likely* (score=10); higher scores reflect a greater likelihood to recommend the program. Ratings between 0 and 6 indicate dissatisfaction and a low likelihood to recommend; ratings of 7 to 8 indicate satisfaction and a moderate likelihood to recommend; and ratings of 9 or 10 indicate high satisfaction and a strong likelihood to recommend to others.

#### CBT Experts

CBT experts completed questionnaires assessing demographics, the SUS, the Likelihood to Recommend scale, and Tranquility’s level of fidelity to CBT protocols for anxiety. They also followed clinical case vignettes through the program to detail the therapeutic components of CBT that are necessary to treat the depicted cases of depression.

##### The Component Analysis of Tranquility

To assess fidelity to CBT components for the treatment of anxiety, we adapted the evaluation criteria for web-based apps treating depression by Huguet et al [13]. We adapted that measure to be specific to the Tranquility program and the treatment of anxiety. The measure includes 10 items, such as “In your opinion, does the Tranquility program provide an explanation of the CBT model?” Responses were rated on a 3-point scale, *none* (score=0), *some* (score=1), and a tailored third option indicating correct and specific use of a therapeutic component (eg, *clear explanation* [score=2]) for each question. All items were summed and divided by the maximum possible score (ie, 20). This value was then multiplied by 100 to create a total score percentage ranging from 0 to 100, with higher scores reflecting greater fidelity to CBT components for treating anxiety.

##### Adaptations to Tranquility

We adapted 2 vignettes of individuals with depression (Multimedia Appendix 1) from the depressive disorders section of the Diagnostic and Statistical Manual of Mental Disorders–5 Clinical Case Handbook [29]—Diane, who has some social anxiety and has been depressed for the last 2 years and experiences little to no interest or pleasure (case 4.6) [30], and Helen, who has been feeling depressed for the last 2 months, drinks 4-5 alcoholic drinks per day, has significant insomnia, had childhood anxiety, and has recently attempted suicide (case 4.10) [31].

CBT experts were asked which CBT components Tranquility should include to offer treatment for depression for the individual in the vignette. Each vignette had the same list of 19 components (eg, behavioral activation and mood tracking; Multimedia Appendix 1). CBT experts were also asked whether there were necessary special considerations when treating individuals seeking treatment for both anxiety and depression.

### Data Collection Procedures

Upon completion of informed consent, all participants were provided with a video link outlining how to access and use Tranquility, and participants were then asked to review Tranquility in detail. To fulfill the 3 aims of the study, data collection occurred in 3 phases. In phase 1, following their review of Tranquility for anxiety, the CBT experts and focus group participants completed measures of usability (ie, SUS) and the likelihood to recommend (ie, Likelihood to Recommend scale). In addition, CBT experts evaluated Tranquility’s treatment fidelity to CBT. All measures were completed on the REDCap (Research Electronic Data Capture; Vanderbilt University) platform, a web-based data collection platform [32,33]. In phase 2, focus group participants participated in a co-design meeting with the researchers using the Zoom Professional platform (Zoom Video Communications Inc); discussions focused on existing anxiety components and adaptations for depression. In phase 3, focus group participants were presented with the survey results from phase 1 to provide a foundation for their feedback. The themes that emerged from the focus group meeting in phase 2 were shown to focus group participants to refine this information and correct any discrepancies. Next, focus group participants were presented with the adaptations made to Tranquility following the previous co-design meeting, and final feedback regarding the adaptations and the integration of anxiety and depression interventions in Tranquility was obtained.

### Data Analysis

As the purpose of the study was not to test a specific hypothesis or compare groups but rather to explore usability, assess treatment fidelity, and engage in the co-design process, data from both groups were pooled for all measures except for CBT fidelity measures only completed by CBT experts. Descriptive statistics were used to summarize the data. Focus group transcripts were analyzed using the principles of thematic content analysis, and we used a data-driven approach to inductively establish themes [34]. The themes were created using the guiding principle of selecting feedback that highlighted the strengths and weaknesses of the Tranquility program. Units of analysis included discrete words, sentences, and paragraphs. In line with the ECOUTER framework, mind maps were used to develop the conceptual schema of themes and subthemes; 2 experienced raters reviewed the anonymized interview transcripts and independently coded the data using Microsoft Excel [35]. Any discrepancies were resolved through discussion.

## Results

### Usability and Likelihood to Recommend

The participants’ ratings of the usability of Tranquility are presented in Table 2. Data were missing from several focus group members (n=4) on measures of usability, resulting in a final sample of 8 participants for the SUS measure. Most participants agreed that Tranquility was easy to use, and all participants agreed with the statement that they felt confident in their ability to use the Tranquility program and that the functions of Tranquility were well-integrated into the program. In contrast, there was less agreement about whether participants would use the program frequently in the future or whether most people would learn to use Tranquility quickly. When looking at reverse-coded items, it was apparent that most participants believed that program users would not need the support of a technical person nor would they need to learn much before being able to use the Tranquility program. Nearly all participants strongly agreed that inconsistency was not a problem across the Tranquility program. The SUS questionnaire results indicated that the average overall usability score was 76.56 (SD 14.07; range 52.5-97.5), which was above the evidence-based cut-off for program usability. Finally, the mean Likelihood to Recommend scale score was 7 (SD 1.07; range 5-8), indicating user satisfaction; 88% (11/12) of participants rated Tranquility as being within the *satisfactory* category, while 1 participant’s rating of Tranquility indicated dissatisfaction and a low likelihood to recommend.

**Table 2:** Usability ratings of the Tranquility program.

Usability component^a^	Rating, mean (SD; range)	Agreement with this statement, n (%)	Disagreement with this statement^b^, n (%)
I think that I would like to use the Tranquility program frequently	3.75 (0.71; 3-5)	5 (63)	N/A^c^
I thought that the Tranquility program was easy to use	4.29 (0.76; 3-5)	6 (86)^d^	N/A
I found the various functions in the Tranquility program were well integrated	4.25 (0.46; 4-5)	8 (100)	N/A
I would imagine that most people would learn to use the Tranquility program very quickly	3.88 (0.99; 2-5)	6 (75)	N/A
I felt very confident using the Tranquility program	4.13 (0.35; 4-5)	8 (100)	N/A
*I needed to learn a lot of things before I could get going with the Tranquility program* ^e^	1.75 (1.04; 1-4)	N/A	7 (86)
*I found the Tranquility program unnecessarily complex* ^e^	2.13 (0.99; 1-4)	N/A	6 (75)
*I think that I would need the support of a technical person to be able to use the Tranquility program* ^e^	1.5 (0.53; 1-2)	N/A	8 (100)
*I thought there was too much inconsistency in the Tranquility program* ^e^	1.75 (0.46; 1-2)	N/A	8 (100)
*I found the Tranquility program cumbersome to use* ^e^	2.13 (0.83; 1-4)	N/A	7 (86)

^a^Ratings ranged from *strongly disagree* (score=1) to *strongly agree* (score=5).

^b^Reverse-coded items.

^c^N/A: not applicable.

^d^There was missing data for 1 participant (n=7).

^e^Items that are reverse-scored (ie, low scores mean higher usability) are italicized.

### Focus Group Content Analysis and Adaptations to Tranquility

#### Content Analysis

Focus group feedback was organized into 8 themes and 8 subthemes (Multimedia Appendix 2). Broadly, feedback from the focus groups focused on suggestions to increase engagement (eg, personalization and reduced psychoeducation) and increase the accessibility of the written content. For the newly added depression material, participants suggested using the word *depression* rather than alternative terms (eg, low mood), ways to tailor the material to a variety of users, the addition of several treatment targets (eg, functioning and self-care), and how to increase the clarity of advertisements about Tranquility.

#### Program Changes

As a part of the co-design process, changes were made to Tranquility across the existing anxiety components and the new depression components (Multimedia Appendix 2). CBT experts’ strong agreement about the necessary components to treat comorbid anxiety and depression resulted in the adaptation of the existing cognitive elements for the treatment of anxiety (eg, thought records and cognitive distortions) for the treatment of depression, and the inclusion of behavioral activation strategies.

The focus group participants suggested providing the program user with more control over their experience and more support for engagement, resulting in increased control over aspects of notifications (eg, notification type), and ongoing developments for Tranquility include gamification and new incentives to foster engagement. Focus group participants asked that coaches initiate coaching appointments, help personalize the messaging experience with clients, and aid users in selecting content that is best suited to their needs. All requested changes applicable to coaching were made to foster connections with the coach and enrich the experience with Tranquility.

The focus group also encouraged the addition of information about program fit before beginning the program; Tranquility now begins with a screening assessment to ensure a good fit between the user’s identified needs and the Tranquility program before payment, and feedback is provided to the user during onboarding. The focus group participants said that there was too much psychoeducation, and the language level was too high for most laypeople; all language was adjusted by increasing layperson terminology and reducing jargon. Finally, the group discussed program tailoring, the addition of treatment targets, and more accurate advertising. As a result, Tranquility can be tailored to begin with either anxiety or depression content, depending on user needs; now includes quality of life and well-being tracking; and is more specifically advertised as a daily use program targeting mild to moderate anxiety and depression.

### CBT Components

CBT experts evaluated the fidelity of Tranquility to CBT for the treatment of anxiety. The average rating for fidelity to core CBT components was 91% (SD 11.14; range 75-100), and half of the CBT experts indicated that Tranquility included 100% of all required CBT components.

In terms of specific components, all 6 experts reported that Tranquility included clear explanations of both anxiety and the CBT model, including cognitive and behavioral techniques, and Tranquility provided formal ratings of anxiety (eg, 0 to 10 scale) to program users. In total, 4 of 5 (80%) CBT experts agreed that Tranquility included specific emotion monitoring, 1 of 5 (20%) therapists agreed that Tranquility included only some emotion monitoring, and 1 expert did not respond. Similarly, 5 of 6 (83%) experts agreed that Tranquility provided specific monitoring of cognitions, and 1 of 6 (17%) therapists indicated that Tranquility included only some monitoring of cognitions. This exact pattern of results was also seen when evaluating whether Tranquility provided a method to monitor behaviors. Only 4 of 6 (67%) CBT experts reported that Tranquility offered a way to monitor specific physical sensations and allowed for adequate case conceptualization of anxiety. For both physical sensation monitoring and case conceptualization, one CBT expert believed there was some tracking of physical sensation monitoring or case conceptualization, while another expert believed there was none.

### Adaptations to Depression Content

#### Common Findings Across Vignettes

All CBT experts indicated that the following components should be included to treat both individuals depicted in the vignettes: psychoeducation about depression, behavioral activation, mood tracking, and case conceptualization. In addition, 5 of 6 (83%) experts agreed that mood ratings (eg, rating mood from 0 to 10), behavioral experiments, and problem-solving skills would also be important to include in Tranquility to treat both cases, while 4 of 6 (67%) experts agreed that coping strategies and sleep hygiene information should be included.

#### Diane Vignette

All 6 (100%) experts agreed that the Diane case would also require pleasant activity scheduling and symptom or outcome tracking. In total, 5 of 6 (83%) experts indicated that it would be helpful to include thought records, while 4 of 6 (67%) experts believed that the identification of cognitive distortions was important to include. Only 2 of 6 (33%) experts indicated that it was important to include motivational interviewing, physical symptom monitoring, substance use tracking, exposure stepladders, or psychoeducation about safety behaviors. Experts also suggested that the following components should also be added: mindfulness, meditation, open journaling, and CBT for insomnia.

#### Helen Vignette

There was unanimous agreement that Helen would benefit from the inclusion of thought records within Tranquility (6/6, 100%). In total, 5 of 6 (83%) experts indicated their belief that pleasant activity scheduling and the identification of cognitive distortions should also be used within Tranquility to treat depression affecting Helen. In total, 4 of 6 (67%) experts believed that substance use tracking, psychoeducation about safety behaviors, and symptom or outcome tracking should also be added. Half of the experts (3/6, 50%) thought motivational interviewing could be beneficial to include, while one-third (2/6, 33%) thought that physical symptom tracking should be included. No CBT expert endorsed the addition of exposure stepladders. Experts also suggested that the following components be added: safety planning, list of crisis resources, mindfulness, meditation, open journaling, and CBT for insomnia.

## Discussion

### Principal Findings

This study represents an assessment of both usability and treatment fidelity to CBT and a co-design adaptation of an iCBT program. Overall, participants highly rated the usability of Tranquility and indicated satisfaction with the program, and CBT experts provided high ratings for Tranquility’s treatment fidelity to CBT. The co-design adaptation process resulted in several improvements to Tranquility.

Experts agreed that Tranquility had high fidelity to the CBT model and included the most necessary components for the treatment of anxiety. There was high agreement across most components (eg, inclusion of behavioral techniques), although to obtain perfect fidelity, Tranquility would need to make emotion, behavior, physical sensation, and cognition monitoring more explicit. These elevated fidelity ratings were not unexpected, as the Tranquility program development team included a licensed clinical psychologist with extensive expertise in CBT. Notably, a recent functionality analysis of apps for depression [36] found that of a possible 8 CBT components, 22% of apps only included ≥3 CBT components, 68% included only 1 or 2, and 10% of apps did not include any CBT components. Furthermore, only 45% of these apps had expert involvement (eg, health professionals) during the app development process. These findings highlight the gap that Tranquility fills with regard to fidelity to CBT components and the importance of fidelity evaluations by objective experts.

Across vignettes, CBT experts typically agreed on which CBT interventions were necessary to treat the depicted cases. Moreover, both cases required the same set of interventions, except for the addition of safety behavior psychoeducation and substance use tracking for the Helen vignette, given the depicted substance use. It was an expected finding that experts would strongly suggest using interventions such as behavioral activation, given its robust efficacy in depression treatment, including within an iCBT format [37]. The integration of therapeutic strategies for depression within an iCBT program for anxiety is likely to be beneficial, given the overlap between the symptoms and the core therapeutic strategies used to treat each disorder [38]. However, the addition of unique therapeutic elements also used to treat either anxiety or depression can overwhelm program users; individuals may benefit from external support to navigate and select the most helpful strategies. A recent literature review [39] suggested that guided (eg, coaches or therapist support) programs had similar rates of adherence as in-person CBT treatment, which is higher than self-guided programs—guidance appears to positively impact treatment program use [39,40].

### Comparison With Prior Work

Current guidelines for digital mental health interventions strongly suggest using a co-design process [41,42], and the results of this study illustrate the importance of that process—the mental health peers suggested crucial improvements to program customizability, delivery, accessibility, and available interventions. Previous research has found that collaborative study designs, such as the co-design approach (see Hill et al [15] for a review), can have a positive effect on aspects such as user adherence, usability, and uptake by involved stakeholders (eg, program users and clinicians).

Mental health programs targeting depression and anxiety need to have both high fidelity to the CBT treatment model and be engaging, flexible, and allow user personalization. Similarly, usability testing is critical, and Kushniruk [43] found that usability testing with small groups (ie, 8-10 users) can drastically reduce the number of usability issues experienced by users in the future. Usability testing within this study revealed areas upon which Tranquility program developers can improve, such as increasing user engagement to encourage users to continue to use this program frequently and reducing the initial learning curve of how to use Tranquility.

The findings of this study are in line with much of the iCBT literature—users want engagement with and control over their web-based therapeutic experiences. Stawarz et al [36] noted that although approximately 58% of CBT apps included at least 1 engagement strategy, they used a less-varied complement of engagement strategies compared with other kinds of mental health apps. Most CBT apps included strategies such as visual aids (eg, graphs and charts) but few included more complex strategies, for example, gamification and coaching or therapist chat functions. Stawarz et al [36] also conducted a qualitative analysis of app reviews, which highlighted the value of leveraging engagement strategies (eg, personalization) to increase user satisfaction with mental health programs [44]. Similarly, a qualitative study of engagement with an iCBT program found that participants wanted to choose what information to learn, accessible content (eg, audiovisual content and appropriate reading level), and a more tailored coaching experience [16,45]. In sum, users want personalized content and more complex engagement strategies.

### Usability of Methodology

The ECOUTER framework provided an accessible approach that centered potential users in the feedback process and provided a procedure guide, which was beneficial when recruiting and working collaboratively with focus group participants as well as during data analysis. Engaging in an adaptation co-design process significantly improved program design and delivery for this iCBT program because it permitted the integration of researchers’ expertise pertaining to the design and delivery of iCBT and participants’ expertise pertaining to mental health both as a clinician and as a client. Moreover, seeking usability feedback from all participants aided in the rigor of this mixed methods evaluation and co-design adaptation of Tranquility because it allowed for multiple perspectives to be considered as CBT experts and focus group members may have different experiences and expectations of iCBT programs and may place value on differing program elements measured within the usability questionnaire we used, the SUS. However, although this approach had many benefits, it did include challenges such as recruiting CBT experts. Many of the approached individuals declined to participate, likely due to demands in excess of their available time, and scheduling focus groups was especially challenging. The usability of the design used in this study, although challenging at times, provides a greater *real-world* approach to mental health program design and both fidelity and usability evaluation.

### Future Directions and Limitations

These findings have therapeutic implications for the iCBT literature. Taken together, future research and iCBT program development should consider the involvement of program users and clinical experts to ensure that fidelity to CBT and engagement strategies are implemented, given its association with adherence and program effectiveness [46]. The effectiveness of gamification and incentives offered within eHealth tools, including within iCBT programs, remains an understudied element of the literature [15], and future work should aim to assess the effectiveness of these user engagement strategies in addition to measuring client satisfaction. In addition, future work is needed to examine the effectiveness of iCBT interventions for comorbid mental health challenges, such as anxiety and depression, and the addition of iCBT protocols to address other challenges (insomnia, trauma, etc) may also help meet treatment demands for other comorbid presentations (eg, anxiety and insomnia).

Similarly, many studies, including this one, included predominantly White, heterosexual women. As a result, there are additional considerations related to race, gender, and sexual orientation that were not captured in the participant feedback or in the resultant changes to Tranquility (eg, using acceptance or values-based strategies vs cognitive restructuring for microaggressions; greater role for family or community members).

Understanding the perspectives and unique needs of marginalized groups is necessary to increase iCBT treatment accessibility and effectiveness—efforts should be made to include participants who are racialized and 2SLGBTQ+ (ie, two-spirit, lesbian, gay, bisexual, transgender, queer, and all other members of this community) and individuals who live in rural areas or are older adults. Efforts to recruit a larger and more diverse sample will afford a greater range of perspectives. Furthermore, this study included a small sample of CBT experts and focus group participants. This smaller sample was a significant strength of the study in that it afforded the focus group participants an opportunity to provide rich and in-depth perspectives on Tranquility and suggestions for adaptation. However, we recognize that the sample size does impact the generalizability of these findings to individuals who were underrepresented in the participant pool (eg, men, nonbinary individuals, or Black people) and who may or may not have been represented at all (eg, people with disabilities). Finally, it is of note that technological confidence and knowledge were not measured; therefore, it is possible that the ratings of this program were influenced by this variable.

### Conclusions

CBT experts and mental health peers agreed that Tranquility, a web-based program treating anxiety and depression, had high usability, and both groups would be likely to recommend this program to others. CBT experts scored Tranquility as having high fidelity to CBT, and nearly all intervention components needed to treat depression were included as a part of Tranquility. Finally, the co-design process was key to refining the existing anxiety content and for the creation and integration of the new depression content. These results provide a preliminary evaluation of the Tranquility program, and they may provide user-centered engagement strategies that may help increase adherence and effectiveness for the iCBT treatment of anxiety and depression.

## Appendix

Multimedia Appendix 1 Cognitive behavioral therapy expert and focus group question list and case vignettes.

Multimedia Appendix 2 Focus group feedback and changes to the Tranquility program.

## Abbreviations

CBT: cognitive behavioral therapy
ECOUTER: Employing Conceptual Schema for Policy and Translation Engagement in Research
iCBT: internet cognitive behavioral therapy
REDCap: Research Electronic Data Capture
SUS: System Usability Scale
2SLGBTQ+: two-spirit, lesbian, gay, bisexual, transgender, queer, and all other members of this community

## References

[1] Hofmann SG, Asnaani A, Vonk IJJ, Sawyer AT, Fang A (2012). The efficacy of cognitive behavioral therapy: a review of meta-analyses. Cognit Ther Res.

[2] Weitz ES, Hollon SD, Twisk J, van Straten A, Huibers MJ, David D, DeRubeis RJ, Dimidjian S, Dunlop BW, Cristea IA, Faramarzi M, Hegerl U, Jarrett RB, Kheirkhah F, Kennedy SH, Mergl R, Miranda J, Mohr DC, Rush AJ, Segal ZV, Siddique J, Simons AD, Vittengl JR, Cuijpers P (2015). Baseline depression severity as moderator of depression outcomes between cognitive behavioral therapy vs pharmacotherapy: an individual patient data meta-analysis. JAMA Psychiatry.

[3] (2020). Trends in the prevalence of depression and anxiety disorders among working-age Canadian adults between 2000 and 2016. Statistics Canada.

[4] Shafran R, Clark DM, Fairburn CG, Arntz A, Barlow DH, Ehlers A, Freeston M, Garety PA, Hollon SD, Ost LG, Salkovskis PM, Williams JM, Wilson GT (2009). Mind the gap: improving the dissemination of CBT. Behav Res Ther.

[5] Wright JH, Mishkind M, Eells TD, Chan SR (2019). Computer-assisted cognitive-behavior therapy and mobile apps for depression and anxiety. Curr Psychiatry Rep.

[6] Dozois DJ (2021). Anxiety and depression in Canada during the COVID-19 pandemic: a national survey. Can Psychol / Psychologie canadienne.

[7] Salari N, Hosseinian-Far A, Jalali R, Vaisi-Raygani A, Rasoulpoor S, Mohammadi M, Rasoulpoor S, Khaledi-Paveh B (2020). Prevalence of stress, anxiety, depression among the general population during the COVID-19 pandemic: a systematic review and meta-analysis. Global Health.

[8] Kumar V, Sattar Y, Bseiso A, Khan S, Rutkofsky IH (2017). The effectiveness of internet-based cognitive behavioral therapy in treatment of psychiatric disorders. Cureus.

[9] Spek V, Cuijpers P, Nyklícek I, Riper H, Keyzer J, Pop V (2006). Internet-based cognitive behaviour therapy for symptoms of depression and anxiety: a meta-analysis. Psychol Med.

[10] Bassett SS, Stein L, Rossi JS, Martin RA (2016). Evaluating measures of fidelity for substance abuse group treatment with incarcerated adolescents. J Subst Abuse Treat.

[11] Perepletchikova F, Treat TA, Kazdin AE (2007). Treatment integrity in psychotherapy research: analysis of the studies and examination of the associated factors. J Consult Clin Psychol.

[12] Bowie-DaBreo D, Sünram-Lea SI, Sas C, Iles-Smith H (2020). Evaluation of treatment descriptions and alignment with clinical guidance of apps for depression on app stores: systematic search and content analysis. JMIR Form Res.

[13] Huguet A, Rao S, McGrath PJ, Wozney L, Wheaton M, Conrod J, Rozario S (2016). A systematic review of cognitive behavioral therapy and behavioral activation apps for depression. PLoS One.

[14] David D, Cristea I, Hofmann SG (2018). Why cognitive behavioral therapy is the current gold standard of psychotherapy. Front Psychiatry.

[15] Hill C, Creswell C, Vigerland S, Nauta MH, March S, Donovan C, Wolters L, Spence SH, Martin JL, Wozney L, McLellan L, Kreuze L, Gould K, Jolstedt M, Nord M, Hudson JL, Utens E, Ruwaard J, Albers C, Khanna M, Albano AM, Serlachius E, Hrastinski S, Kendall PC (2018). Navigating the development and dissemination of internet cognitive behavioral therapy (iCBT) for anxiety disorders in children and young people: a consensus statement with recommendations from the #iCBTLorentz Workshop Group. Internet Interv.

[16] Yardley L, Spring BJ, Riper H, Morrison LG, Crane DH, Curtis K, Merchant GC, Naughton F, Blandford A (2016). Understanding and promoting effective engagement with digital behavior change interventions. Am J Prev Med.

[17] Sartorius N, Üstün TB, Lecrubier Y, Wittchen H (2018). Depression comorbid with anxiety: results from the WHO study on psychological disorders in primary health care. Br J Psychiatry.

[18] Pencer A, Tucker R, Muise J (2019). Breaking down barriers: evaluating an internet-based CBT program for adults experiencing anxiety. Proceedings of the 9th World Congress of Behavioural and Cognitive Therapies.

[19] Pencer A, Tucker R, Muise J, Wozney L (2020). Breaking down barriers and optimizing retention: a novel approach to delivering iCBT. Proceedings of the Poster Presentation at the 9th Annual E-mental Health Conference.

[20] Thüring M, Mahlke S (2007). Usability, aesthetics and emotions in human–technology interaction. Int J Psychol.

[21] Bevan N (1995). Measuring usability as quality of use. Software Qual J.

[22] Canadian Institutes of Health Research, Natural Sciences and Engineering Research Council of Canada, Social Sciences and Humanities Research Council (2018). Tri-Council Policy Statement: Ethical Conduct for Research Involving Humans – TCPS 2 (2018).

[23] Murtagh MJ, Minion JT, Turner A, Wilson RC, Blell M, Ochieng C, Murtagh B, Roberts S, Butters OW, Burton PR (2017). The ECOUTER methodology for stakeholder engagement in translational research. BMC Med Ethics.

[24] Brooke J, Jordan P, Thomas B, Weerdmeester B (1996). SUS: A 'Quick and Dirty' usability scale. Usability Evaluation In Industry.

[25] Bangor A, Kortum PT, Miller JT (2008). An empirical evaluation of the system usability scale. Int J Hum-Comput Interact.

[26] Lewis JR (2018). The system usability scale: past, present, and future. Int J Hum–Comput Interact.

[27] Mol M, van Schaik A, Dozeman E, Ruwaard J, Vis C, Ebert DD, Etzelmueller A, Mathiasen K, Moles B, Mora T, Pedersen CD, Skjøth MM, Pensado LP, Piera-Jimenez J, Gokcay D, Ince BU, Russi A, Sacco Y, Zanalda E, Zabala AF, Riper H, Smit JH (2020). Dimensionality of the system usability scale among professionals using internet-based interventions for depression: a confirmatory factor analysis. BMC Psychiatry.

[28] Reichheld FF (2003). The one number you need to grow. Harv Bus Rev.

[29] Barnhill J, Wood W, Yonkers K (2013). Depressive disorders. DSM-5 Clinical Cases.

[30] Brody B (2013). Depressive disorders case 4.6. DSM-5 Clinical Cases: Depressive Disorders.

[31] Goldberg J (2013). Depressive disorders case 4.10. DSM-5 Clinical Cases.

[32] Harris PA, Taylor R, Thielke R, Payne J, Gonzalez N, Conde JG (2009). Research electronic data capture (REDCap)--a metadata-driven methodology and workflow process for providing translational research informatics support. J Biomed Inform.

[33] Harris PA, Taylor R, Minor BL, Elliott V, Fernandez M, O'Neal L, McLeod L, Delacqua G, Delacqua F, Kirby J, Duda SN, REDCap Consortium (2019). The REDCap consortium: building an international community of software platform partners. J Biomed Inform.

[34] Braun V, Clarke V (2006). Using thematic analysis in psychology. Qualitative Research in Psychology.

[35] Bree RT, Gallagher G (2016). Using Microsoft Excel to code and thematically analyse qualitative data: a simple, cost-effective approach. All Ireland J High Edu.

[36] Stawarz K, Preist C, Tallon D, Wiles N, Coyle D (2018). User experience of cognitive behavioral therapy apps for depression: an analysis of app functionality and user reviews. J Med Internet Res.

[37] Furukawa TA, Suganuma A, Ostinelli EG, Andersson G, Beevers CG, Shumake J, Berger T, Boele FW, Buntrock C, Carlbring P, Choi I, Christensen H, Mackinnon A, Dahne J, Huibers MJ, Ebert DD, Farrer L, Forand N, Strunk DR, Ezawa ID, Forsell E, Kaldo V, Geraedts A, Gilbody S, Littlewood E, Brabyn S, Hadjistavropoulos HD, Schneider LH, Johansson R, Kenter R, Kivi M, Björkelund C, Kleiboer A, Riper H, Klein JP, Schröder J, Meyer B, Moritz S, Bücker L, Lintvedt O, Johansson P, Lundgren J, Milgrom J, Gemmill AW, Mohr DC, Montero-Marin J, Garcia-Campayo J, Nobis S, Zarski A, O'Moore K, Williams AD, Newby JM, Perini S, Phillips R, Schneider J, Pots W, Pugh NE, Richards D, Rosso IM, Rauch SL, Sheeber LB, Smith J, Spek V, Pop VJ, Ünlü B, van Bastelaar KM, van Luenen S, Garnefski N, Kraaij V, Vernmark K, Warmerdam L, van Straten A, Zagorscak P, Knaevelsrud C, Heinrich M, Miguel C, Cipriani A, Efthimiou O, Karyotaki E, Cuijpers P (2021). Dismantling, optimising, and personalising internet cognitive behavioural therapy for depression: a systematic review and component network meta-analysis using individual participant data. Lancet Psychiatry.

[38] Păsărelu CR, Andersson G, Bergman NL, Dobrean A (2017). Internet-delivered transdiagnostic and tailored cognitive behavioral therapy for anxiety and depression: a systematic review and meta-analysis of randomized controlled trials. Cogn Behav Ther.

[39] Radomski AD, Wozney L, McGrath P, Huguet A, Hartling L, Dyson MP, Bennett K, Newton AS (2019). Design and delivery features that may improve the use of internet-based cognitive behavioral therapy for children and adolescents with anxiety: a realist literature synthesis with a persuasive systems design perspective. J Med Internet Res.

[40] Wozney L, Huguet A, Bennett K, Radomski AD, Hartling L, Dyson M, McGrath PJ, Newton AS (2017). How do eHealth programs for adolescents with depression work? A realist review of persuasive system design components in internet-based psychological therapies. J Med Internet Res.

[41] Achilles MR, Anderson M, Li SH, Subotic-Kerry M, Parker B, O'Dea B (2020). Adherence to e-mental health among youth: considerations for intervention development and research design. Digit Health.

[42] Scariot CA, Heemann A, Padovani S (2012). Understanding the collaborative-participatory design. Work.

[43] Kushniruk A (2002). Evaluation in the design of health information systems: application of approaches emerging from usability engineering. Comput Biol Med.

[44] Batterham PJ, Calear AL, Farrer L, McCallum SM, Cheng VW (2018). FitMindKit: Randomised controlled trial of an automatically tailored online program for mood, anxiety, substance use and suicidality. Internet Interv.

[45] Walsh A, Richards D (2016). Experiences and engagement with the design features and strategies of an internet-delivered treatment programme for generalised anxiety disorder: a service-based evaluation. Br J Guid Counsell.

[46] Hilvert-Bruce Z, Rossouw PJ, Wong N, Sunderland M, Andrews G (2012). Adherence as a determinant of effectiveness of internet cognitive behavioural therapy for anxiety and depressive disorders. Behav Res Ther.

