# Supplementary material for: An Internet-Based Cognitive Behavioral Therapy Program for Anxiety and Depression (Tranquility): Adaptation Co-design and Fidelity Evaluation Study
Source: JMIR Form Res. 2022 Feb 2;6(2):e33374. doi: 10.2196/33374 (PMC8851319; doi:10.2196/33374)
Supplement: Multimedia Appendix 1 [file formative_v6i2e33374_app1.doc]

# Multimedia Appendix

This is a Multimedia Appendix to a full manuscript published in the J Med Internet Res. For full copyright and citation information see <http://dx.doi.org/10.2196/33374>

## CBT vignettes

### Vignette 1

(Adapted from DSM-5 Clinical Case Handbook, case 4.6) [29]

Diane, a 29-year-old laboratory technician, has sought help from the Tranquility program at the suggestion of her work supervisor who noticed she became tearful while being mildly criticized during an otherwise positive annual performance review. Somewhat embarrassed, she told her supervisor that she had been “feeling low for years” and that hearing criticism of her work had been “just too much.”

She felt frustrated with her job, which she saw as a “dead end,” and struggled with guilty feelings that she “hadn’t done much” with her life. Diane feels like she enjoys life a lot less in the past few years and isn’t interested in very many things.

Despite her troubles at work, Diane felt that she could concentrate without difficulty. She denied ever having active suicidal thoughts, yet sometimes wondered, “What is the point of life?” She reported that she sometimes had trouble falling asleep but often felt very fatigued in the morning and throughout the day. She did not report any changes to her weight, but her appetite had significantly decreased. Although she occasionally would go out with coworkers, she said that she felt very shy and awkward in social situations unless she knew the people well. She noted that her symptoms waxed and waned but had remained consistent over the past 2 years. She had no symptoms of mania or hypomania. There is a family history of depression on her mother’s side.

Diane first became depressed when she was in high school, shortly after her father was hospitalized for leukemia. She has no other psychiatric or medical history. She revealed that she had been sexually abused by a family friend during her childhood and has had consistent dysfunctional romantic relationships and currently has few friends.

#### Questions

1. What therapeutic components should Tranquility include to offer treatment for depression for Diane? (check all that apply)
   - Motivational interviewing
   - Behavioural activation
   - Psychoeducation about depression
   - Ratings of current level of depressed mood (i.e., 0 to 10 scale)
   - Thought records
   - Mood tracking
   - Case conceptualization
   - Pleasant activity scheduling
   - Physical symptom monitoring
   - Substance use tracking
   - Coping strategy tracking
   - Identification of cognitive distortions
   - Exposure stepladders
   - Behavioural experiments
   - Psychoeducation about safety behaviours
   - Problem solving skills
   - Sleep hygiene information
   - Symptom/outcome tracking (e.g., questionnaires)
   - Other
     1. Please describe: __________________________________

### Vignette 2

(Adapted from DSM-5 Clinical Case Handbook, case 4.10) [30]

Helen, a 27-year-old administrative assistant, has sought help for her depression from the Tranquility program. She recently broke up with her romantic partner of 2 years. She said she had been feeling increasingly sad and hopeless for 1–2 months in anticipation of the breakup. She recently attempted to overdose on pain relief medication and later felt immense guilt for this attempt. She has never experienced thoughts of suicide prior to this attempt.

During these past 8 weeks, Helen’s mood had been moderately depressed throughout the day most days, but her mood could improve temporarily if something happy happened. She had recently gained about 10 pounds from “overeating comfort food and junk.” She described her self-esteem as “non-existent” and has found it hard to feel motivation or to concentrate on daily tasks. She struggles to fall asleep and will toss and turn all night until 4-5am and then wake at 7am and “trudge through the day.” She said she will sometimes drink wine in the evening to help her feel sleepy and feel better; she said she “doesn’t drink much, only 4-5 glasses of wine.”

Helen saw a counselor when she was in her first year of university because she was feeling “moody and low” and was struggling to focus in class. At that time, she began escitalopram and psychotherapy, but improved quickly and stopped both after a few weeks. Growing up, Helen said she was a “quiet, anxious” child and “not a troublemaker.” Her older brother abused multiple substances, although Helen said she herself had never used illegal drugs. Helen’s younger sister had frequent “panic attacks and depression.”

#### Questions

1. What therapeutic components should Tranquility include to offer treatment for depression for Helen? (check all that apply)
   - Motivational interviewing
   - Behavioural activation
   - Psychoeducation about depression
   - Ratings of current level of depressed mood (i.e., 0 to 10 scale)
   - Thought records
   - Mood tracking
   - Case conceptualization
   - Pleasant activity scheduling
   - Physical symptom monitoring
   - Substance use tracking
   - Coping strategy tracking
   - Identification of cognitive distortions
   - Exposure stepladders
   - Behavioural experiments
   - Psychoeducation about safety behaviours
   - Problem solving skills
   - Sleep hygiene information
   - Symptom/outcome tracking (e.g., questionnaires)
   - Other
     1. Please describe: _________________________________

## Focus group questions

1. **Think about the in-app features of Tranquility - which of these matters most? What do you think would make a difference for you or other people even wanting to start it?**
   - 1. Email notifications
     2. In-app notifications
     3. Coaching
     4. Routine monitoring and feedback
     5. Interactive tools
2. **If you were to use this program and choose to have reminders to complete tasks, would you want to pick your own schedule, have them once a week, or some other schedule?**
3. **Now thinking of what depression looks like for people - what kinds of things would be important to track? Some ideas we had were tracking mood or substance use (like alcohol use).**
4. **After seeing our mock-up of how we would integrate depression treatment into Tranquility, is there anything missing that you think needs to be there?**
5. **What do you think is *the* most important thing that needs to be on Tranquility for treating depression? Which part do you think is going to be the most beneficial?**
6. **Imagine you don’t know much about depression but came to Tranquility to learn more and to get help. What kinds of things would you want to learn about or know about depression?**
7. How much would you want to know about the neuroscience of depression? What about the factors contributing to the development of depression?
8. What are the top 3 things you would want to know about depression??
9. What term is best to use – “depression”, “low mood”, or something else?
10. **Let’s talk about role of the coach. Should their role be different for people who want to work on anxiety compared to those who want to work on depression? What kind of differences should there be?**
11. **Who should schedule coaching – the client or the coach? When should coaching be scheduled?**
12. **What is the main appeal of having both anxiety and depression integrated within the same program?**

10. **What is the biggest drawback to having both anxiety and depression integrated within the same program?**

11. **If you had the choice, would you prefer two different programs, one for anxiety and one for depression? Why?**
