# Supplementary material for: An Internet-Based Cognitive Behavioral Therapy Program for Anxiety and Depression (Tranquility): Adaptation Co-design and Fidelity Evaluation Study
Source: JMIR Form Res. 2022 Feb 2;6(2):e33374. doi: 10.2196/33374 (PMC8851319; doi:10.2196/33374)
Supplement: Multimedia Appendix 2 [file formative_v6i2e33374_app2.docx]

# Multimedia Appendix

This is a Multimedia Appendix to a full manuscript published in the J Med Internet Res. For full copyright and citation information see <http://dx.doi.org/10.2196/33374>

**Table 1.** Identified themes of focus group feedback and related changes made to the Tranquility program.

| Themes and feedback | | | | Changes made to the Tranquility program |
| --- | --- | --- | --- | --- |
| **Engagement** | | | | |
|  | **Supports for engagement** | | |  |
|  |  | | - Use email notifications as reminders - Users want motivational incentives (eg, notification celebrating 3 consecutive days of use and completing a planned exposure) - Want increased control over   notification settings (eg, timing, in-app or email)   - “Journey” emails—updates to program, knowledge applicable to all users, and information about strategies that are most effective for users   Participant quotes:   - “I hate having to figure out if need to unsubscribe in email or go into app to turn this off. I think that would be great thing to add at the beginning. Have an option ‘how would you like to be contacted?’” (P4) - “I think that’s a great idea—not just app notifications, like reminders you have one more thing to do you’re not doing, but communication that’s more positive in nature, more affirming, like ‘great job with that thing you did last week’.” (P5) | - Notifications and reminders for activities were revised and added where applicable - Gamification and incentives to motivate users are currently being developed - User control over whether to receive notifications has been added to the activity tools |
|  | **Barriers to engagement** | | |  |
|  |  | | - Too much psychoeducation—some people may already have information; may delay learning skills and symptom improvement - Currently, users must work hard to remain engaged—they must seek out coaching and keep themselves motivated on their own   Participant quote(s):   - “I agree with the wordiness of the page, even with the anxiety pages, and I think there should be options to be like ‘click here if you want to learn more about signs and symptoms’, but if someone who knows they have anxiety and have already researched it themselves, they may want to flash through these pages, like ‘how will this help me, this will give me skills, this might make me feel better’ instead of having to read it all over again, cause their doctor, or GP, or research on their own, they could already know all this, it’s pretty repetitive and you could find this info anywhere.” (P4) | - Added drop-down menus with optional additional information that interested users can read - Coaching protocols have been redesigned to increase contact and engagement with users |
| **Coaching** | | | | |
|  | **Personal connection** | | |  |
|  |  | | - User reaches out to the coach for help—could be too difficult for some users, suggestion for the coach to schedule coaching upfront - Message from the coach to user is stock template—suggestion to personalize messages based on user responses during program sign-up - Coaching by the same person consistently and tailored check-ins would be helpful   Participant quote(s):   - “I got like a very generic message as soon as coach was assigned, like ‘Hey, this is how this basically works; send a message, and I’ll reply within 24 hours. Here is a Calendly link if you wanna book time with me. Sometimes I’ll look at work to see where you’re at’... templatey for sure. It felt hard... positive that there is support for sure... but also I wish this was structured from patient care perspective ...like, what is this person going through, and how do we proactively support them or create comfy enviro for them to know they have a champion or someone in their corner. I don’t know what that looks like, but I think a little bit more structure to the coaching environment could just maybe just alleviate those pressures or not make you feel as vulnerable or exposed in trying to establish that relationship while you’re also trying to work through some really heavy content” (P1) | - Upon onboarding, the first and only option for users is to message coach or book an appointment. Coaches will reach out if no appointment is made within 24 hours of onboarding - Initial message from the coach remains automated, but the coach reaches out within 24 hours with a personalized message - Coaches continue to provide tailored check-ins with program users |
|  | **Mechanics** | | |  |
|  |  | | - Offer regular coaching sessions (eg, once a week) - Ask program users amount of coaching they want upfront (eg, a little or moderate) - Could be helpful to set goals before meeting coach - Coaching piece will be strengthened by combining depression and anxiety help in one place—easier to alleviate symptoms. There may be a greater need for the role of coach to help users work through program and select most relevant techniques   Participant quote(s):   - “I think the coaching piece is one that will make it stronger by having it all in one place. If I’m working with a coach on anxiety who is also trained, equipped, and knowledgeable about dealing with depression as well in my situation it’s an easy way to help alleviate those pieces as well.” (P2) - “Be clear about what you can offer, what can the coaches offer, what is going to be realistic for customizations. Give folks choices at the beginning and then check in—how is this working? More, or less support? Constant conversations around how they are supporting you, ‘are you finding this useful’ etc.” (P5) | - Coaches are trained to discuss coaching needs with users (eg, amount and frequency of meetings) in their first meeting - Users are encouraged via messaging from coach to complete session 1 and consider making their own SMART^b^ goals - Coaches have been trained to work with the user to select CBT^a^ elements most relevant to their symptoms of anxiety and/or depression |
| **Program fit** | | | | |
|  | - Increase clarity about the kind of user for whom this program is designed (ie, mild to moderate anxiety and depression) - Assess symptoms before beginning program—if severity level too high, can suggest other options to potential users - If user not improving after a period, suggest other resources (eg, visit a physician to discuss medication options)   Participant quote(s):  • “What comes to mind with depression—when first getting introduced to the program, I think it’s important to assess where people might be at with depression. If I’ve been dealing with depression all my life and never identified or acknowledged it, or a life event, like a post-partum situation, or a significant change in life experience, like I lost my job and haven’t been able to find work for 3 months, and now I’m sliding down that road, versus someone where something like an antidepressant might be needed. Like I’ve been dealing with depression all my life and it’s not life event triggered, to me, that feels different and what the platform can do for me might be different in those different situations so, um, letting people have an opportunity before—cause essentially people are purchasing this, so the diagnosis or understanding what this is going to do for me shouldn’t happen after I’ve already put down my credit card, right, it should happen beforehand. I think that’s important to keep in mind as well and then communicating that to the end user, so ‘if this is your profile, it’ll be most effective for you’, you want to avoid trying the program and it not being the right fit for them.” [P2] | | | - New onboarding has several messages and feedback opportunities about the purpose of Tranquility and fit for the user - Symptoms are assessed before sign-up to ensure the program is the right fit for the user - Onboarding includes information about other options for users if symptom severity is deemed too high or complex during onboarding to benefit from Tranquility |
| **Safety** | | | | |
|  | - Include a disclaimer on messages with the coach (eg, “If you’re experiencing a crisis, contact a physician or access your local ER”) or include an “I’m in crisis” button that redirects the user to a list of immediately accessible resources - Ensure coaches are trained on suicide prevention and discussing suicide and self-harm   Participant quote(s):   - “I think it would be important that coaches were trained on suicide prevention, having some understanding what the conversation might look like when it’s past a coaching role, and being comfortable to ask the question ‘do you think you might hurt yourself’ so coaches are in more of a position, working with depression to have to—they’re not going to be counselling and that’s past the role of a coach. Like in community suicide prevention education, we talk about having the conversation and then helping to direct people where they need to go. That might not come across as much in the anxiety program would but would when you’re dealing with depression.” (P2) - “We want to make sure if we’re redirecting someone to resources, the resources are going to appropriately respond. My concern is always—legal issue—my concern is always if we say to people ‘call this number’, but the crisis number isn’t working now because it's off hours, is that appropriate? Don’t send people to a dead end.” (P5) | | | - Disclaimer that “If you’re experiencing a crisis, contact a physician or access your local ER” is now on every page of the program, including on the coach messaging board - Coaches trained to discuss suicide and self-harm and to direct users to 911 or local emergency, if necessary |
| **Health literacy** | | | | |
|  | **Mental health literacy** | | |  |
|  |  | | - Anxiety concepts were difficult to understand at times—required a high level of mental health literacy - May not be necessary to understand all anxiety-related cognitive distortions and instead focus on those most relevant to the user - Depression is not always triggered by a life event—could have no identifiable trigger or be related to biological changes (eg, postpartum and menstrual cycle hormonal changes) - Increase normalization of depression - Reduce psychoeducation about depression; focus on the messages that “depression is treatable,” “there is hope,” and “Tranquility uses effective, evidence-based techniques”   Participant quote(s):   - “By the time they’ve arrived at your website, they’ve already identified that something is not right, and they want to take action, so I don’t think you need to explain ‘depression can be really lame’ because they are already there. So, think of what events landed them on this page and what do you need to do to move them to the next step with this.” (P5) - “Maybe ‘there’s hope’, that’s session 1, ‘you’re gonna be okay’ is the message.” (P1) - “It might be worth exploring how much do you need to apply or understand the information [cognitive distortions information]. Like for everything that’s there, maybe as a client, you don’t need to understand it all, I need to understand how it impacts me.” (P2) | - There is a drop-down menu within the cognitive distortion section allowing users to choose those relevant to them - Updated psychoeducation about depression—clarifies that depression is not necessarily caused by a life event - Normalization of depression done through Personas (ie, stories of people experiencing depression) - Psychoeducation focuses on the usefulness of CBT for depression |
|  | **Reading level** | | |  |
|  |  | | - Website information is written at a high reading level - Proposed depression information is written at a high level—make information more accessible with layperson terms   Participant quote(s):   - “I found the content to be a little complex or advanced. I have studied psychology and I’m familiar with mental filters, etc. But even me going through them, it was complex, the wording. I was wondering at what level that language was geared? Although it’s clinically wonderful and evidence based, I’m worried you’ll lose people because it’s a bit hard to understand for someone without that literacy.” (P3) - “It’s a lot of text here. You’ve got a really high reading level, just to be mindful of that.” (P5) | - Reviewed language and literacy level and all information, including new depression content, is now written at a grade 8 level or lower |
|  | **Terminology** | | |  |
|  |  | | - Use the term “depression.” Others said that “low mood” is not a layperson term—“depression” and “anxiety” are terms used by laypeople - Consider depression with a lowercase “d” and an uppercase “D” to distinguish normative changes in mood compared with clinically significant declines in mood - Feature that logs activities during the day—suggested names were activity log, mood log, and life log. Life log was perceived negatively; if few activities, a user may say “I have no life”   Participant quote(s):   - “My concern with low mood is that we know what that generally means, but I think that to the lay community, we know depression, we know anxiety. I don’t think the term ‘low mood’ means something to those that are not familiar with that language.” (P1) - “[Discussing in-app daily activity tracker proposed name—'life log’] ‘I’m a 3/10 out of life’. So, it’s almost like ‘I suck at life’? ...like it’s just the way it’s just the word ‘life’. And you don’t want everyone’s life to revolve around their mood, especially if they’re just like, ‘wow my life just sucks because I have a 2/10 all the time’.” (P4) | - Using the terms “depression” and “low mood” throughout Tranquility—users identify with both terms - Given increased focus on layperson terminology and clarity, chose to use “low mood” as descriptor for nonclinical depression and depression to indicate clinical depression - User suggested “Activity Log” label for the activity recording feature |
| **Tailoring** | | | | |
|  | - Program stream selection (self-directed vs coaching) is helpful—offers client choice and can choose the journey that is best fit for the user - May need adaptations depending on type of depression (eg, chronic, postpartum) - Not everyone experiences sadness during depression—could be irritability, poor sleep, or decreased energy that alerts them to the problem - The integration of anxiety and depression may be overwhelming and/or hard to navigate for some users. Yet, this integration is critical, given the common co-occurrence of anxiety and depression - Allow for the creation of own values rather than selecting from a stock list—may better cater to the needs of a variety of users   Participant quote(s):   - “I love the idea of building user-driven choice into setup part—‘I’m self-directed, I want to do it this way’, they can choose that path, or they find person-to-person coaching more supportive—any opportunity to allow the user to chat about what they need and map out what level of interaction they want—daily, weekly email, in-app alerts, coach to email them once a week; building in those options would be helpful.” (P5) | | | - Users can choose a self-guided or coach-guided experience for anxiety and depression - Have added 4 personas with different levels and types of depression to help broaden the applicability to more users - Users start with anxiety or depression. Users will have several automated “check-ins”—Tranquility (program and/or coach) may recommend additional content for anxiety or depression based on their Generalized Anxiety-7 (GAD-7) measure and Patient Health Questionnaire-9 (PHQ-9) measure scores and users can decide what to add to their program. |
| **Treatment targets** | | | | |
|  | - Participants appreciated that Tranquility can track mood to see if and why your mood improved or worsened - Could track other aspects during depression (eg, well-being and quality of life) - Suggested to track self-care in new depression components (eg, social time or personal grooming) - Ask about functioning, that is, ability to go to school or work. Track functioning ratings and use as evidence of improvement—may also be an important target for users   Participant quote(s):  • “I love having measures around overall well-being, quality of life, self-compassion—I like measures around that. [...] So, we’re not just asking ‘How much does your life suck right now?’ but questions that are framed in a more positive way.” (P5)  • “I think an option to include a section when you recognize that something really upset you or made you excited and lifted your mood, an honest journal on—how to word it—highs and lows! So, you can look back and say, ‘That really upset me then but now I know that wouldn’t upset me now if it happened again.’ Not from clinical perspective so much, it’s just from the client’s perspective. For depression, reliving scenarios can help you overcome that for some clients.” (P4) | | | - Quality of life is now tracked biweekly using the mini Quality of Life Enjoyment and Satisfaction Questionnaire [32] - Well-being is tracked before beginning the program and after finishing the Tranquility program using the longer version of the mini Quality of Life Enjoyment and Satisfaction Questionnaire (Q-LES-Q-SF) [33] |
| **Advertising** | | | | |
|  | - Tranquility should be advertised as a daily maintenance program rather than as a program accessed only when feeling unwell   Participant quote(s):   - “I feel like that’s where Tranquility could come in and it could be like health management. So, it’s not necessarily for people to go to when they’re feeling like ‘I need to log onto Headspace and talk to something’, but something about maintenance where they go to it every day and sustain their health because I think that’s really important for a lot of these mental health apps. Not just going to it when you’re upset, but to help people stay on the baseline of feeling good and checking in always and feeling different on different days but knowing that it’s something that they should do every day.” (P4) | | | - Tranquility is advertised as a daily use program to help with mild to moderate anxiety and depression |

^a^CBT: cognitive behavioral therapy.

^b^SMART goals: Specific, Measurable, Achievable, Relevant, Time-bound goals
